# Fibroblast growth factor 23 as a biomarker of right ventricular dysfunction in pulmonary hypertension

**DOI:** 10.1007/s00392-023-02162-y

**Published:** 2023-02-15

**Authors:** Laila Widmann, Stanislav Keranov, Leili Jafari, Christoph Liebetrau, Till Keller, Christian Troidl, Steffen Kriechbaum, Sandra Voss, Mani Arsalan, Manuel J. Richter, Khodr Tello, Henning Gall, Hossein A. Ghofrani, Stefan Guth, Werner Seeger, Christian W. Hamm, Oliver Dörr, Holger Nef

**Affiliations:** 1 Department of Cardiology and Angiology, University of Giessen, Klinikstr. 33, 35392 Giessen, Germany; 2 DZHK (German Center for Cardiovascular Research), Partner Site RheinMain, Bad Nauheim, Germany; 3 Department of Cardiology, Kerckhoff Heart and Lung Center, Bad Nauheim, Germany; 4 Cardioangiological Center Bethanien (CCB), Frankfurt, Germany; 5 Department of Internal Medicine, Justus-Liebig-University Giessen, Universities of Giessen and Marburg Lung Center (UGMLC), Institute for Lung Health (ILH), Cardio-Pulmonary Institute (CPI), Member of the German Center for Lung Research (DZL), Giessen, Germany; 6 Department of Thoracic Surgery, Kerckhoff Heart and Lung Center, Bad Nauheim, Germany

**Keywords:** IPAH, CTEPH, RV-PA coupling, Fibrosis, DCM, LV hypertrophy

## Abstract

**Background:**

Fibroblast growth factor 23 (FGF-23) has been associated with left ventricular hypertrophy (LVH) and heart failure. However, its role in right ventricular (RV) remodeling and RV failure is unknown. This study analyzed the utility of FGF-23 as a biomarker of RV function in patients with pulmonary hypertension (PH).

**Methods:**

In this observational study, FGF-23 was measured in the plasma of patients with PH (*n* = 627), dilated cardiomyopathy (DCM, *n* = 59), or LVH with severe aortic stenosis (*n* = 35). Participants without LV or RV abnormalities served as controls (*n* = 36).

**Results:**

Median FGF-23 plasma levels were higher in PH patients than in healthy controls (*p* < 0.001). There were no significant differences between PH, DCM, and LVH patients. Analysis across tertiles of FGF-23 levels in PH patients revealed an association between higher FGF-23 levels and higher levels of NT-proBNP and worse renal function. Furthermore, patients in the high-FGF-23 tertile had a higher pulmonary vascular resistance (PVR), mean pulmonary artery pressure, and right atrial pressure and a lower cardiac index (CI) than patients in the low tertile (*p* < 0.001 for all comparisons). Higher FGF-23 levels were associated with higher RV end-diastolic diameter and lower tricuspid annular plane systolic excursions (TAPSE) and TAPSE/PASP. Receiver operating characteristic analysis revealed FGF-23 as a good predictor of RV maladaptation, defined as TAPSE < 17 mm and CI < 2.5 L/min/m^2^. Association of FGF-23 with parameters of RV function was independent of the glomerular filtration rate in regression analysis.

**Conclusion:**

FGF-23 may serve as a biomarker for maladaptive RV remodeling in patients with PH.

**Graphic abstract:**

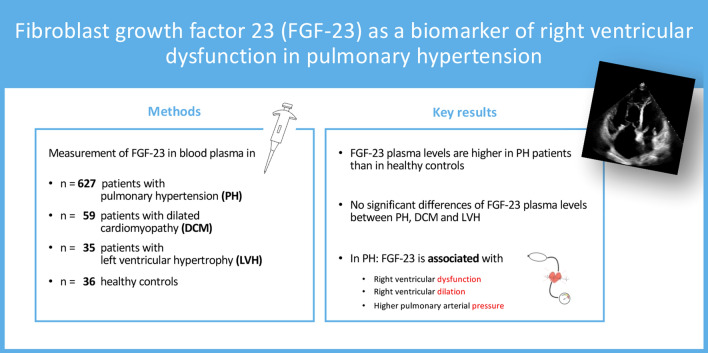

**Supplementary Information:**

The online version contains supplementary material available at 10.1007/s00392-023-02162-y.

## Introduction

Pulmonary hypertension (PH) increases the afterload of the right ventricle (RV). Chronic pressure overload leads to RV remodeling and may result in right heart failure, which is associated with adverse outcomes [2]. Maladaptive RV remodeling is characterized by dilation, excessive fibrosis, and systolic and diastolic dysfunction [36]. Early detection of maladaptive RV remodeling in PH is crucial for the prevention of RV failure, but it remains challenging. Hence, biomarkers indicating maladaptive changes in RV function could be useful noninvasive diagnostic tool to optimize the treatment of PH patients.

Fibroblast growth factor 23 (FGF-23) is a bone-derived protein that acts as a regulator in phosphate and vitamin D homeostasis and is elevated in patients with chronic kidney disease (CKD) [27]. A direct effect of FGF-23 on the heart to induce hypertrophy and fibrosis was shown in vitro [5, 12]. In clinical studies elevated FGF-23 was associated with left ventricular (LV) hypertrophy and was shown to be a strong predictor of cardiovascular mortality in patients with heart failure and reduced (HFrEF) or preserved (HFpEF) LV ejection fraction [5, 9, 25, 28].

To date, there is a paucity of data on the role of FGF-23 in RV remodeling. A recent study reported an association between high serum levels of FGF-23 and pulmonary arterial hypertension (PAH) in a small cohort of patients undergoing dialysis [20]. Interestingly, another study with HFpEF patients showed an association between increased FGF-23 plasma levels and parameters of maladaptive RV remodeling in echocardiography and cardiac magnetic resonance imaging (CMR), whereas there was no association between LV parameters and FGF-23 levels [28]. These results suggest that FGF-23 could have differential expression patterns in LV and RV remodeling.

The aim of the present study was to examine the utility of FGF-23 as a biomarker of RV dysfunction in a large cohort of PH patients. Furthermore, FGF-23 plasma levels were compared in patients with PH, dilated cardiomyopathy (DCM), left ventricular hypertrophy (LVH) in severe aortic stenosis, and controls without any LV or RV abnormalities.

## Methods

### Study population

Between December 2013 and January 2021, *n* = 542 patients with chronic thromboembolic pulmonary hypertension (CTEPH), *n* = 85 with idiopathic pulmonary hypertension (IPAH), *n* = 35 patients with LVH and severe aortic stenosis, and *n* = 59 DCM patients were enrolled in this observational cohort study. Individuals (*n* = 34) without any RV or LV abnormalities served as controls. Study inclusion took place at the Kerckhoff Heart and Thorax Center and at the University Hospital of Giessen and Marburg (UKGM) Campus Gießen, Department of Cardiology.

Data on demographics, comorbidities, and symptoms were collected for all patients. RV and LV function were evaluated by transthoracic echocardiography, and patients with pulmonary hypertension were also examined by right heart catheterization (RHC, see below).

All patients gave written informed consent before enrollment in this study. The Institutional Review Board of the University of Giessen approved the study (199/15, 44/14, 100/13).

### Inclusion criteria

Patients with IPAH and CTEPH were included if they fulfilled all of the following criteria: age > 18 years, mean pulmonary artery pressure (mPAP) ≥ 25 mmHg, pulmonary artery wedge pressure (PAWP) ≤ 15 mmHg, left ventricular ejection fraction (LVEF) ≥ 50%, and no LVH (end-diastolic interventricular septum thickness (IVSd) ≤ 13 mm).

Patients with LVH and pressure overload were included in the study according to the following criteria: chronic LV pressure overload due to severe aortic stenosis (aortic valve mean pressure gradient > 40 mmHg and/or aortic valve area < 1 cm^2^) and preserved LV function (LVEF > 55%), IVSd > 12 mm.

Patients diagnosed with DCM were included in the study according to the following criteria: impaired LV function (LVEF < 40%), LVEDd > 56 mm as measured by echocardiography, at the time of diagnosis.

Samples from a cohort without any RV or LV abnormalities were used as controls. Chronic kidney disease was defined as an estimated glomerular filtration rate (eGFR) < 60 mL/min/1.73 m^2^. eGFR was estimated using the CKD-EPI method.

### Laboratory assessments

Venous blood samples were obtained by venipuncture at inclusion and collected in plain tubes. Plasma aliquots were processed immediately and stored at − 80 °C until further analysis. Biomarker measurements were performed by experienced staff who were blinded to patients’ characteristics.

C-terminal FGF-23 was measured by a second-generation human FGF-23 enzyme-linked immunosorbent assay (ELISA) kit (MicroVue Human FGF-23 (C-Term) EIA-96 Test, Quidel Corporation, Athens, Ohio, USA) with a minimum detectable concentration of 1.5 RU/mL. The intra-assay coefficients of variation are 2.4% and 1.4% at 33.7 and 302 RU/mL, and the respective interassay coefficients of variation are 4.7% and 2.4% at 33.6 and 293 RU/mL.

In a subset of patients (*n* = 335) NT-pro-BNP levels were measured in serum with an electrochemiluminescence immunosassay using monoclonal antibodies (NT-proBNP assay, Roche Diagnostics, Mannheim, Germany). The intra-assay coefficients of variation are 1.5% and 1.3% at 124 and 14.142 pg/mL, respectively, and the respective interassay coefficients of variation are 2.7% and 1.7% at 125 and 32,930 pg/mL as declared by the package insert. The lower detection limit for the NT-proBNP assay is 5 pg/mL.

### Transthoracic echocardiography

Transthoracic two-dimensional echocardiography was performed in all patients according to international recommendations [17]. Right heart function was examined using tricuspid annular plane systolic excursion (TAPSE), right ventricular end-diastolic diameter (RVEDD), pulmonary artery systolic pressure (PASP), and the ratio of TAPSE to PASP (TAPSE/PASP). TAPSE/PASP is an index that takes into account both the shortening (TAPSE) and the developed strength (PASP) of the RV [10], which has been shown to be an important prognostic parameter in PH [32].

### Right heart catheterization (RHC)

RHC was performed in standard fashion via the right internal jugular vein using a 6F sheath and a standard Swan-Ganz catheter. Medication was not changed prior to or during the procedure. No vasoactive substances were administered.

### Statistical analysis

Continuous variables are shown as mean ± standard deviation or as median with interquartile range, as appropriate. Categorial variables are displayed as numbers and percentages. The Shapiro–Wilk test was applied to assess parametric distribution. Independent cohorts were compared using Student’s *t* test for normally distributed variables or the Mann–Whitney *U* test for non-normally distributed continuous variables. The Kruskal–Wallis test with Dunnet’s post-hoc test was applied to compare non-normally distributed variables in more than two subgroups. The Chi-squared test and Fisher’s exact test were used for categorical variables. Receiver operating characteristic (ROC) curve analysis was performed to assess the predictive value of FGF-23 regarding RV maladaptation, defined as TAPSE < 17 mm and cardiac index (CI) < 2.5 L/min/m^2^. Binary logistic regression analysis was performed to identify whether FGF-23 is a glomerular filtration rate (GFR)-independent predictor of parameters of RV function. A two-tailed *p* value < 0.05 was considered to define statistical significance. Statistical analysis was performed using IBM SPSS Statistics Version 28.0 (IBM Corp., Armonk, NY, USA).

## Results

### Characteristics of the study population

Clinical characteristics of patients with PH, LVH, DCM, and controls are shown in Table 1. Patients with LVH were older, had the lowest estimated GFR (eGFR) and had a higher rate of atrial fibrillation, coronary artery disease, and previous percutaneous coronary intervention, coronary artery bypass surgery, and myocardial infarction than patients in the other groups (*p* < 0.05 for all comparisons). PH patients had higher PASP measured by echocardiography than that of all other groups. In addition, the echocardiographic RV parameters TAPSE and TAPSE/PASP were lower in PH patients than in the other groups, whereas RVEDD was higher (*p* < 0.05 for all comparisons).

**Table 1:** Clinical characteristics

	PH	LVH	DCM	Controls
	*n* = 627	*n* = 35	*n* = 59	*n* = 34
Baseline characteristics				
Female sex, *n*	299 (47.7%)	19 (54.3%)	17 (28.8%)	17 (50.0%)
Age, years, mean (SD)	60 (46–74)	81 (76–86)	58 (45–71)	52 (32–72)
BMI, kg/m^2^, mean (SD)	28 (22–34)	26 (22–30)	30 (23–37)	26 (22–30)
NYHA ≥ III	299 (77.7%)	19 (55.9%)	25 (43.1%)	3 (8.8%)
Medical history				
Atrial fibrillation, *n*	56 (10.3%)	18 (51.4%)	0 (0.0%)	1 (2.9%)
CAD, *n*	90 (14.4%)	25 (71.4%)	10 (16.9%)	11 (32.4%)
Previous MI, *n*	19 (3.5%)	12 (34.3%)	0 (0.0%)	3 (8.8%)
Previous PCI, *n*	24 (5.9%)	16 (45.7%)	0 (0.0%)	9 (26.5%)
Previous CABG, *n*	1 (0.2%)	3 (8.6%)	0 (0.0%)	1 (2.9%)
PAD, *n*	10 (2.5%)	2 (5.7%)	0 (0.0%)	2 (5.9%)
Previous stroke/TIA, *n*	14 (3.5%)	0 (0.0%)	0 (0.0%)	2 (5.9%)
Cardiovascular risk factors				
Diabetes mellitus, *n*	65 (10.4%)	8 (22.9%)	12 (20.7%)	2 (5.9%)
Hypertension, *n*	241 (50.1%)	29 (82.9%)	29 (50.0%)	12 (35.3%)
Dyslipidemia, *n*	82 (17.3%)	14 (40.0%)	15 (25.9%)	9 (26.5%)
Family history of CV disease, *n*	55 (14.2%)	11 (33.3%)	24 (44.4%)	11 (32.4%)
smoking, *n*	69 (11.8%)	2 (5.9%)	25 (44.6%)	6 (17.6%)
Right heart catheterization				
PVR, dyn/s/cm^3^, median (IQR)	544 (404–728)	n. a	n. a	n. a
PAPmean, mmHg, median (IQR)	42 (35–49)	n. a	20 (17–27)	n. a
PAWPmean, mmHg, median (IQR)	9 (7–12)	n. a	15 (10–21)	n. a
RAP, mmHg, median (IQR)	7 (5–10)	n. a	5 (3–8)	n. a
CI, L/min/m^2^, median (IQR)	2.4 (2.0–2.8)	n. a	1.9 (1.7–2.4)	n. a
Echocardiography				
PASP, mmHg, median (IQR)	78 (63–92)	33 (26–37)	28 (24–34)	26 (22–27)
TAPSE, mm, median (IQR)	19 (16–23)	21 (19–23)	20 (18–22)	24 (22–29)
RVEDd, mm, median (IQR)	44 (39–51)	36 (34–41)	37 (34–41)	38 (35–40)
LV-EF, %, median (IQR)	60 (55–60)	55 (45–60)	30 (20–35)	60 (55–60)
IVSd, mm, median (IQR)	10 (9–11)	13 (11–15)	11 (9–12)	10 (9–11)
LVPWd, mm, median (IQR)	10 (8–11)	12 (10–13)	11 (9–11)	11 (10–11)
*E*/*E*′, median (IQR)	9 (8–13)	21 (16–25)	15 (11–20)	7 (7–10)
LA, mm, median (IQR)	33 (29–39)	44 (39–50)	44 (40–50)	36 (34–37)
LVEDd, mm, median (IQR)	44 (40–47)	48 (41–54)	62 (57–67)	46 (41–48)
TAPSE/PASP, mm/mmHg, median (IQR)	0.25 (0.18–0.34)	0.61 (0.54–0.82)	0.71 (0.55–0.87)	0.89 (0.83–0.89)
Biomarkers				
Creatinine, mg/dL, median (IQR)	0.9 (0.8–1.1)	1.1 (0.9–1.3)	1.0 (0.8–1.2)	0.8 (0.7–1.0)
GFR, mL/min/1.73 m^2^, median (IQR)	79 (64–97)	63(42–86)	81 (62–101)	94 (70–114)
FGF-23, RU/mL, median (IQR)	98 (67–133)	105 (66–151)	88 (46–114)	46 (2–82)

*PH* pulmonary hypertension, *LVH* left ventricular hypertrophy, *DCM* dilated cardiomyopathy, *BMI* body mass index, *NYHA* New York Heart Association, *CAD* coronary artery disease, *PCI* percutaneous coronary intervention, *CABG* coronary artery bypass grafting, *PAD* peripheral artery disease, *TIA* transient ischemic attack, *CV* cardiovascular, *PVR* pulmonary vascular resistance, *PAPmean* mean pulmonary artery pressure, *PAWPmean* mean pulmonary artery wedge pressure, *RAP* right atrial pressure, *CI* cardiac index, *PASP* pulmonary artery systolic pressure, *TAPSE* tricuspid annular plane systolic excursion, *RVEDD* right ventricular enddiastolic diameter, *LVEF* left-ventricular ejection fraction, *IVSd* diastolic interventricular septum thickness, *LVPWd* diastolic left ventricular posterior wall thickness, *LA* left atrium, *LVEDD* left ventricular enddiastolic diameter, *GFR* glomerular filtration rate, *FGF-23* fibroblast growth factor 23

### FGF-23 levels

Clinical characteristics of patients with PH, LVH, DCM, and controls are shown in Table 1.

**Fig. 1:**
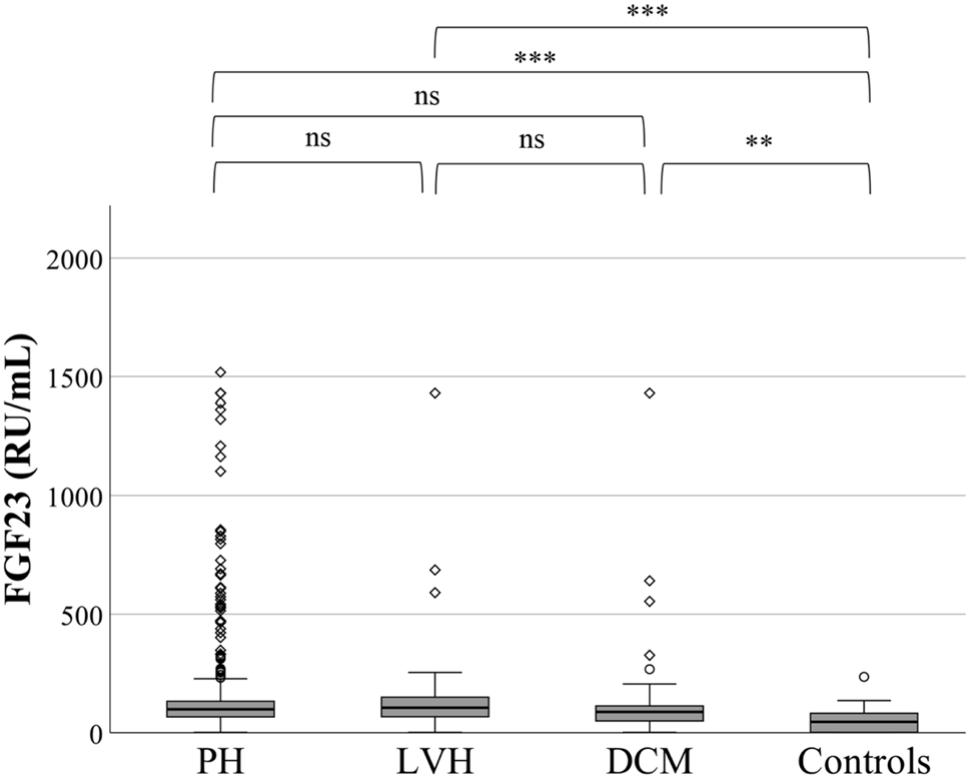
Box plots comparing FGF-23 levels in patients with PH, LVH, DCM and in controls. Boxes represent median with IQR. *p < 0.05, **p < 0.01, ***p < 0.001. *ns* not significant, *FGF23* fibroblast growth factor 23, *PH* pulmonary hypertension, *LVH* left ventricular hypertrophy, *DCM* dilated cardiomyopathy

Median FGF-23 plasma levels were significantly higher in patients with PH, DCM, or LVH than in healthy controls (*p* < 0.01 for all comparisons), whereas there were no significant differences between PH, DCM, and LVH patients (Fig. 1). In patients with LVH, FGF-23 plasma levels correlated with age (*r* = 0.43, *p* = 0.01) and eGFR (*r* = − 0.43, *p* = 0.01). No significant correlation was observed between FGF-23 levels and echocardiographic parameters of LV or RV function. (Table 2). In patients with DCM, FGF-23 levels did not correlate with age (*r* = − 0.03, *p* = 0.34) but did correlate with eGFR (*r* = − 0.29, *p* = 0.03), RVEDD (*r* = 0.27, *p* = 0.04), TAPSE (*r* = − 0.43, *p* < 0.001), TAPSE/PASP (− 0.35, *p* = 0.01), mPAP (*r* = 0.51, *p* = 0.003), and LVEF (*r* = − 0.31, *p* = 0.02). No correlation was observed between FGF-23 and right atrial pressure (RAP, *r* = 0.12, *p* = 0.15), cardiac index (CI, *r* = − 0.1, *p* = 0.21), LVEDd (*r* = − 0.12, *p* = 0.13) IVSd (0.10, *p* = 0.14) or LVPWd (0.12, *p* = 0.11) in DCM patients (Table 3).

**Table 2:** Correlation of FGF-23 with clinical and echocardiographic characteristics in patients with LVH

	Pearson correlation	*p* value
Age	− 0.43	0.01
GFR	− 0.43	0.01
RVEDD	0.18	ns
TAPSE	− 0.14	ns
TAPSE/PASP	− 0.27	ns
LVEF	0.01	ns
LVEDD	0.26	ns
LVPWd	0.04	ns
IVSd	− 0.25	ns

*FGF-23* fibroblast growth factor 23, *LVH* left ventricular hypertrophy, *GFR* glomerular filtration rate, *RVEDD* right ventricular enddiastolic diameter, *TAPSE* tricuspid annular plane systolic excursion, *PASP* pulmonary artery systolic pressure, *LVEF* left-ventricular ejection fraction, *LVEDD* left ventricular enddiastolic diameter, *LVPWd* diastolic left ventricular posterior wall thickness, *IVSd* diastolic interventricular septum thickness

**Table 3:** Correlation of FGF-23 with clinical, echocardiographic and invasively measured characteristics in patients with DCM

	Pearson correlation	*p* value
Age	− 0.03	ns
GFR	− 0.29	0.03
RVEDD	0.27	0.039
TAPSE	− 0.43	< 0.001
TAPSE/PASP	− 0.35	0.01
LVEF	− 0.31	0.02
LVEDD	− 0.12	ns
LVPWd	0.10	ns
IVSd	0.12	ns
mPAP	0.51	0.003
PAWPmean	0.56	ns
RAP	0.12	ns
CI	− 0.10	ns

*FGF-23* fibroblast growth factor 23, *DCM* dilated cardiomyopathy, *GFR* glomerular filtration rate, *RVEDD* right ventricular end diastolic diameter, *TAPSE* tricuspid annular plane systolic excursion, *PASP* pulmonary artery systolic pressure, *LVEF* left-ventricular ejection fraction, *LVEDD* left ventricular end diastolic diameter, *LVPWd* diastolic left ventricular posterior wall thickness, *IVSd* diastolic interventricular septum thickness, *mPAP* mean pulmonary artery pressure, *PAWPmean* mean pulmonary artery wedge pressure, *RAP* right atrial pressure, *CI* cardiac index

An analysis of clinical and imaging parameters across FGF-23 tertiles in patients with PH is shown in Table 4. No differences were observed between FGF-23 tertiles regarding mean age, BMI, NYHA class, and comorbidities except for diabetes: patients in the high-FGF-23 tertile showed a higher prevalence of diabetes than those in the low tertile (*p* = 0.04).

**Table 4:** Clinical characteristics of patients with pulmonary hypertension divided into tertiles of FGF-23

	Tertile 1	Tertile 2	Tertile 3	Tertile 3 versus Tertile 1
	(< 78 RU/mL)	(78–118 RU/mL)	(> 118 RU/mL)	
	*n* = 209	*n* = 209	*n* = 209	*p* value
Baseline characteristics				
Female sex, *n*	86 (41.1%)	99 (47.4%)	114 (54.5%)	0.006
Age, years, mean (± SD)	60 (47–73)	60 (45–75)	61 (47–75)	0.71
BMI, kg/m^2^, mean (± SD)	28 (22–34)	28 (22–34)	28 (21–35)	0.87
NYHA functional classes III and IV, *n*	95 (74.2%)	93 (74.4%)	111 (84.1%)	0.05
Medical history				
Atrial fibrillation, *n*	14 (7.5%)	18 (10.1%)	24 (13.5%)	0.06
CAD, *n*	29 (14.0%)	26 (12.5%)	35 (16.8%)	0.43
Previous myocardial infarction, *n*	10 (5.4%)	2 (1.1%)	7 (3.9%)	0.50
Previous PCI, *n*	8 (5.3%)	7 (5.1%)	9 (7.6%)	0.46
Previous CABG, *n*	0 (0.0%)	1 (0.7%)	0 (0.0%)	n. a
PAD, *n*	2 (1.3%)	3 (2.2%)	5 (4.3%)	0.25
Previous stroke/TIA, *n*	3 (2.0%)	9 (6.8%)	2 (1.7%)	1.00
Cardiovascular risk factors				
Diabetes mellitus, *n*	17 (8.2%)	17 (8.1%)	31 (14.8%)	0.04
Hypertension, *n*	80 (47.1%)	80 (48.8%)	81 (55.1%)	0.15
Dyslipidemia, *n*	32 (19.2%)	26 (16.0%)	24 (16.4%)	0.53
Family history of CV disease, *n*	21 (14.4%)	17 (13.4%)	17 (15.0%)	0.88
Smoking, *n*	20 (10.2%)	20 (10.3%)	29 (14.9%)	0.16
Right heart catheterization				
PVR, dyn/s/cm^3^, median (IQR)	447 (324–580)	529 (420–724)	655 (466–858)	< 0.001
PAPmean, mmHg, median (IQR)	39 (32–45)	40 (35–48)	46 (40–52)	< 0.001
PAWPmean, mmHg, median (IQR)	10 (7–12)	9 (7–11)	11 (8–12)	0.37
RAP, mmHg, median (IQR)	6 (5–9)	7 (5–9)	9(6–12)	< 0.001
CI, L/min/m^2^, median (IQR)	2.5 (2.2–2.9)	2.4 (2.0–2.7)	2.2 (1.9–2.7)	< 0.001
Echocardiography				
PASP, mmHg, median (IQR)	69 (57–82)	79 (60–90)	84 (69–98)	< 0.001
TAPSE, mm, median (IQR)	21 (18–24)	20 (16–23)	17 (14–21)	< 0.001
RVEDd, mm, median (IQR)	43 (36–47)	44 (40–49)	50 (43–53)	< 0.001
LV-EF, %, median (IQR)	60 (55–60)	60 (55–60)	60 (55–61)	0.37
IVSd, mm, median (IQR)	10 (9–11)	10 (9–11)	10 (9–12)	0.31
LVPWd, mm, median (IQR)	9 (9–11)	10 (9–10)	10 (8–11)	0.78
LA, mm, median (IQR)	35 (32–38)	31 (27–38)	34 (30–44)	0.69
LVEDd, mm, median (IQR)	46 (42–49)	43 (40–47)	40 (36–46)	0.002
TAPSE/PASP, mm/mmHg, median (IQR)	0.30 (0.22–0.39)	0.26 (0.19–0.34)	0.21 (0.15–0.29)	< 0.001
Creatinine, mg/dL, median (IQR)	0.9 (0.7–1.0)	0.9 (0.8–1.1)	1.0 (0.8–1.3)	< 0.001
GFR, mL/min/1.73 m^2^, median (IQR)	88 (76–102)	79 (65–98)	68 (53–89)	< 0.001
BNP, pg/mL, median (IQR)	51 (24–94)	91 (48–196)	353 (77–525)	0.001
nt-pro-BNP (*n* = 335), ng/L, median (IQR)	308 (115–855)	547 (166–1703)	1712 (700–3253)	< 0.001
FGF-23, RU/mL, median (IQR)	50 (2–67)	99 (88–106)	163 (133–266)	< 0.001

*BMI* body mass index, *NYHA* New York Heart Association, *CAD* coronary artery disease, *PCI* percutaneous coronary intervention, *CABG* coronary artery bypass grafting, *PAD* peripheral artery disease, *TIA* transient ischemic attack, *CV* cardiovascular, *PVR* pulmonary vascular resistance, *PAPmean* mean pulmonary artery pressure, *PAWPmean* mean pulmonary artery wedge pressure, *RAP* right atrial pressure, *CI* cardiac index, *PASP* pulmonary artery systolic pressure, *TAPSE* tricuspid annular plane systolic excursion, *RVEDD* right ventricular enddiastolic diameter, *LVEF* left-ventricular ejection fraction, *IVSd* diastolic interventricular septum thickness, *LVPWd* diastolic left ventricular posterior wall thickness, *LA* left atrium, *LVEDD* left ventricular enddiastolic diameter, *GFR* glomerular filtration rate, *BNP* brain natriuretic peptide, *FGF-23* fibroblast growth factor 23

**Fig. 2:**
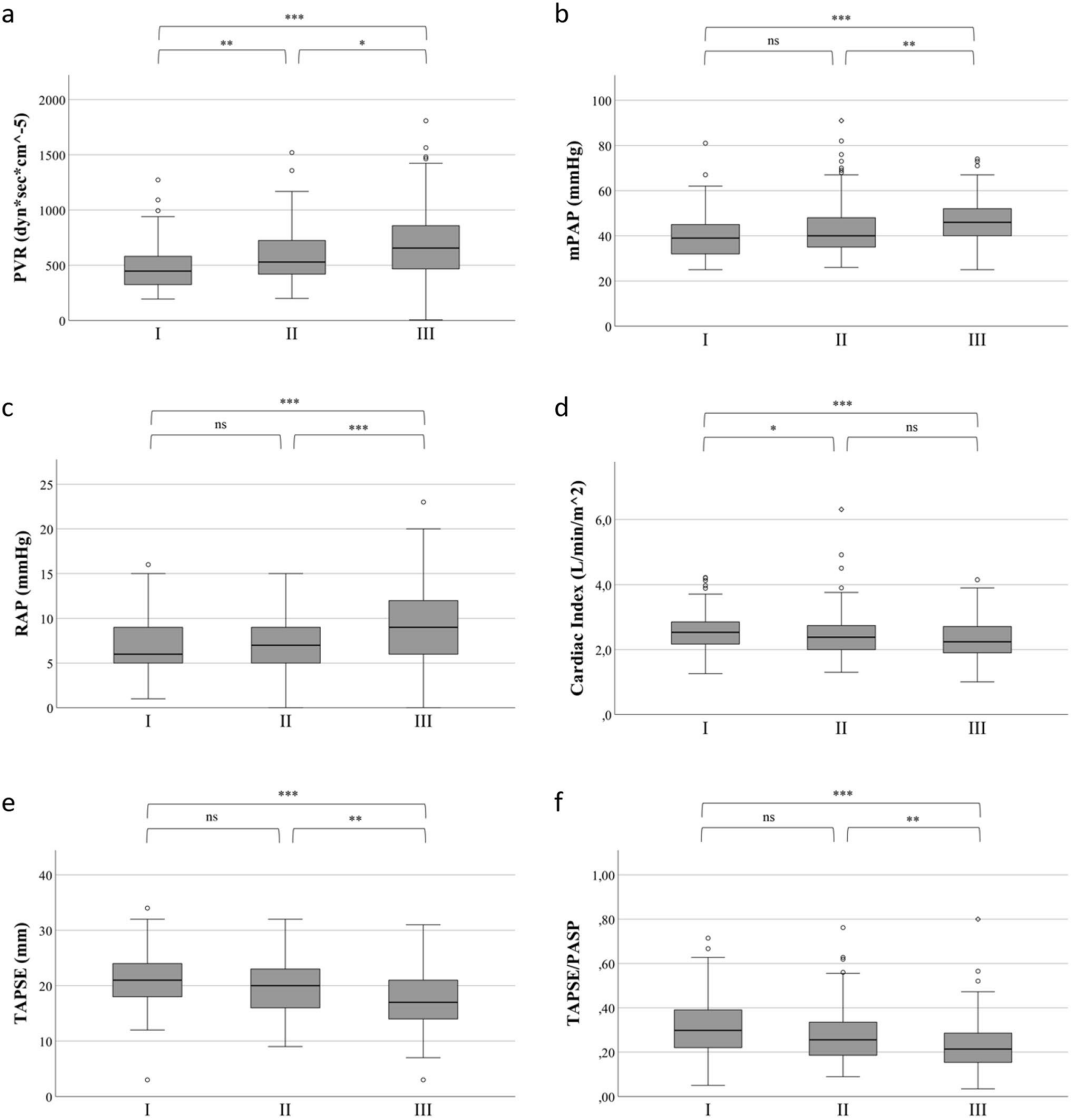

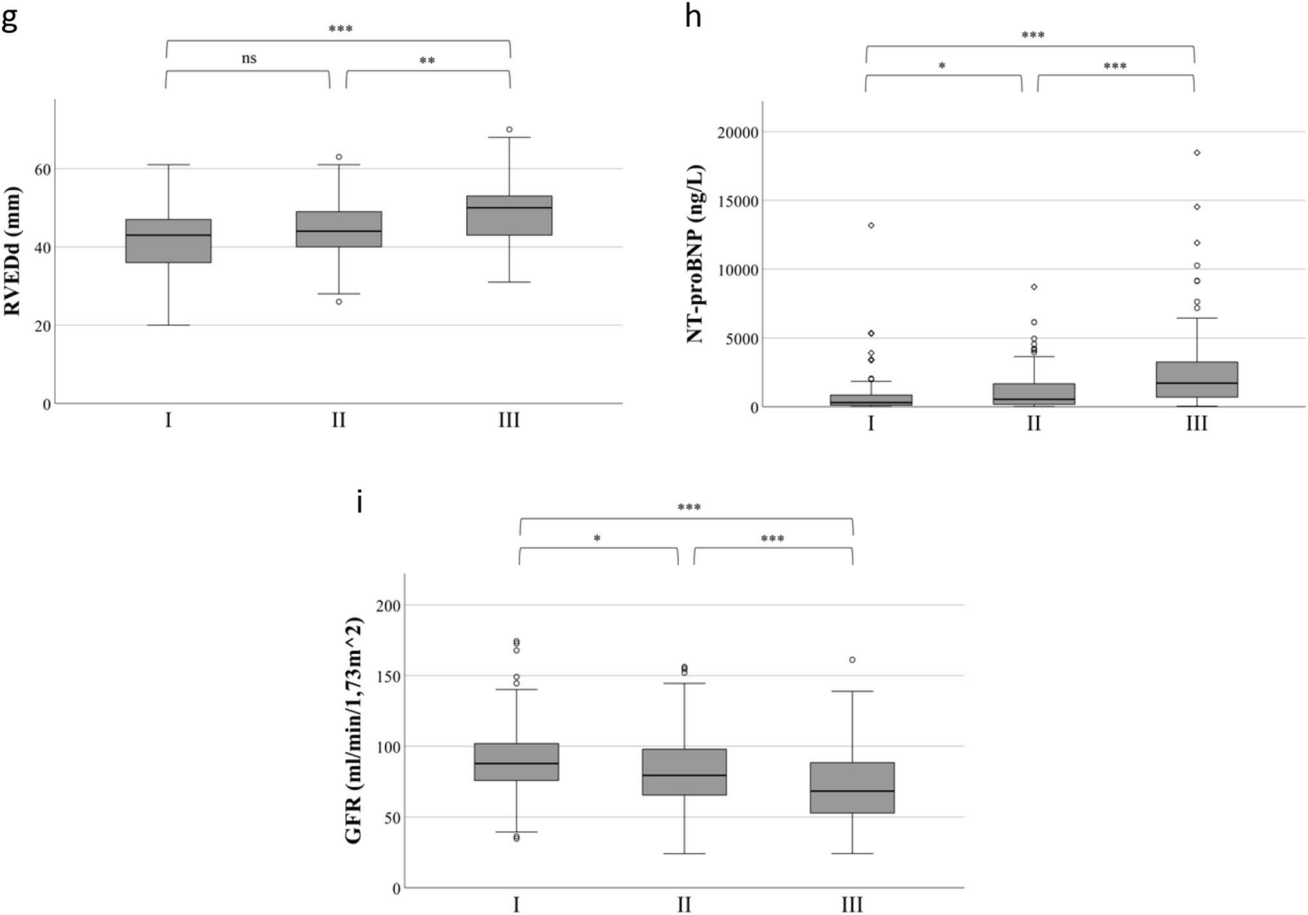
Boxplots showing **a** PVR, **b** mPAP, **c** RAP, **d** Cardiac Index, **e** TAPSE, **f** TAPSE/PASP, **g** RVEDD, **h** NT-proBNP, **i** GFR across FGF-23 tertiles in patients with pulmonary hypertension. I: Low: < 78 RU/mL; II: middle: 78–117 RU/mL; III: high: > 117 RU/mL; boxes represent median with IQR. *FGF-23* fibroblast growth factor 23, *PVR* pulmonary vascular resistance, *mPAP* mean pulmonary artery pressure, *RAP* right atrial pressure, *TAPSE* tricuspid annular plane systolic excursion, *RVEDD* right ventricular enddiastolic diameter, *BNP* brain natriuretic peptide, *GFR* glomerular filtration rate, ***p < 0.001; **p < 0.01; *p < 0.05; *ns* not significant

Assessment of RHC parameters showed that PVR (Fig. 2a), mPAP (Fig. 2b), and RAP (Fig. 2c) were higher in the high-FGF-23 tertile than in the middle (*p* < 0.05 for all comparisons) or the low tertile (*p* < 0.001 for all comparisons). The CI was higher in the low-FGF-23 tertile than in the middle (*p* < 0.05) or high tertile (*p* < 0.001; Fig. 2d). The echocardiographic parameters TAPSE and TAPSE/PASP were lower in patients in the high-FGF-23 tertile than in the middle or low tertile (*p* < 0.01 for all comparisons; Fig. 2e, f). Furthermore, in patients in the high tertile, RVEDD was higher than in patients in the middle or low tertile (*p* < 0.01 for both comparisons; Fig. 2g). NT-proBNP levels in the high-FGF-23 tertile were also higher than in the middle or low tertiles (*p* < 0.001 for both comparisons, Fig. 2h).

eGFR was lower in the high-FGF-23 tertile compared with the middle and low tertiles (*p* < 0.05 for all comparisons, Fig. 2i). The results of binary regression analysis indicated that FGF-23 is an eGFR-independent predictor of TAPSE < 17 mm, CI < 2.0 l/min/m^2^, RVEDD > 43 mm, and RAP > 7 mmHg (Suppl. Table 1). Additionally, correlation analysis in the subgroup of PH patients with CKD (*n* = 93) confirmed the findings from the entire PH cohort (Suppl. Table 2).

**Fig. 3:**
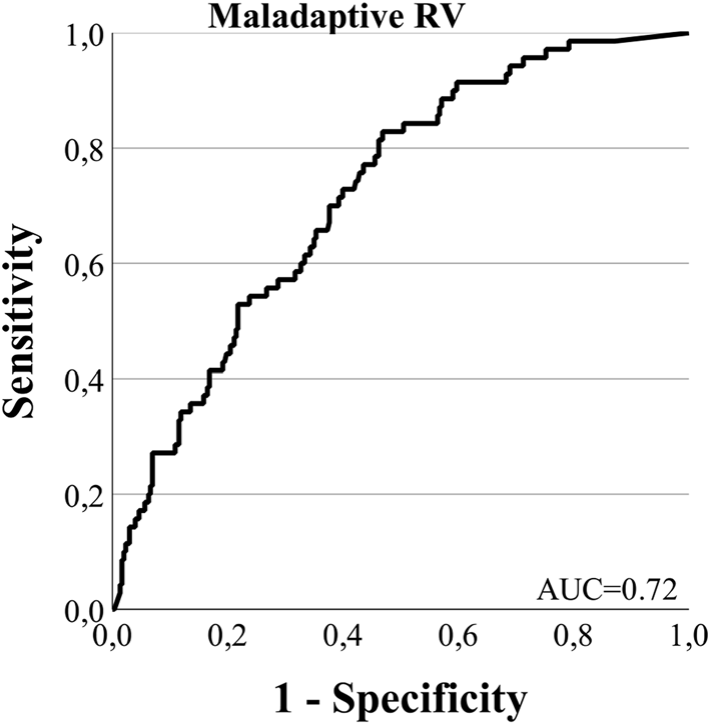
Receiver operating characteristics curve showing the predictive power of FGF-23 for maladaptive RV (TAPSE < 17 mm and CI < 2.5 L/min/m^2^). *FGF-23* fibroblast growth factor 23, *eGFR* estimated glomerular filtration rate, *RV* right ventricle, *TAPSE* tricuspidal annular plane systolic excursion, *CI* cardiac index

ROC analysis identified FGF-23 as a good predictor of maladaptive RV-defined TAPSE < 17 mm and CI < 2.5 L/min/m^2^ in patients with PH (AUC_FGF-23_ = 0.72, Fig. 3).

## Discussion

### Primary study findings

The present study examined the relationship between FGF-23 levels and clinical characteristics, echocardiographic parameters, and invasively measured RHC parameters in patients with PH, LVH, DCM, and in healthy controls. The main findings of this study are that (1) plasma levels of FGF-23 are higher in patients with PH, LVH, and DCM than in healthy controls, whereas no significant differences were found between patients with PH, LVH, and DCM; (2) increased FGF-23 plasma levels are associated with systolic RV dysfunction, RV dilation, lower CI, and higher pulmonary pressure and vascular resistance in PH patients; (3) FGF-23 is an eGFR-independent predictor of RV maladaptation.

### The transition from adaptation to maladaptation of the RV in PH.

The continuous pressure overload caused by PH induces remodeling processes in the RV. In early-stage PH, the RV adapts to its higher afterload by a four to fivefold increase in contractility to maintain cardiac output. This increase is achieved by hypertrophy and changes in cardiomyocyte contractile properties that enable ventriculoarterial coupling to be maintained [37].

Chronic progressive pressure overload, however, leads to increased wall stress, capillary rarefication, and neurohumoral activation and other pathological stimuli that induce maladaptive remodeling in the RV. These changes lead to RV dilation, fibrosis, and systolic and diastolic dysfunction [35]. As a result, the RV can no longer sustain an adequate cardiac output, which leads to ventriculoarterial uncoupling and finally right heart failure, which is associated with high mortality [2].

The early detection of maladaptive changes could be essential for the improvement of therapeutic strategies in patients with pulmonary hypertension. Currently, the gold-standard method for evaluating right ventricular–pulmonary artery (RV–PA) coupling is the measurement of the pressure–volume (PV) loop-derived end-systolic RV elastance/pulmonary elastance (Ees/Ea) ratio [22, 37]. However, this invasive measurement requires a high level of technical expertise and is thus not suitable for routine clinical practice. Non-invasive tools would be very useful for monitoring RV coupling and especially the transition to RV–PA uncoupling. A combination of clinical examination, non-invasive imaging techniques, and biomarker measurements would be a conceivable solution for better patient risk stratification.

### FGF-23 as a biomarker of myocardial remodeling

FGF-23, which is mainly secreted by osteocytes, plays an important role in phosphate and vitamin D homeostasis [31]. In early-stage CKD, FGF-23 levels increase, to keep phosphate levels within the normal range [14]. Beyond its secretion by osteocytes, FGF-23 was discovered to be expressed in cardiac myocytes and cardiac fibroblasts under pathologic conditions such as myocardial infarction, LV pressure overload and heart failure [19]. Furthermore, FGF-23 was involved in profibrotic and prohypertrophic myocardial signaling in several experimental studies [5, 8, 12, 19]. These observations suggest, that FGF-23 plays a role in the pathophysiology of myocardial hypertrophy and myocardial fibrosis.

Our findings that FGF-23 plasma levels were increased in patients with LVH and DCM compared with healthy controls are consistent with previous studies [15, 21, 30]. Patients with DCM also showed significant correlations of FGF-23 levels to parameters of LV and RV remodeling. Patients with LVH, however, did not show any correlations to parameters of myocardial remodeling. Adversely, findings from previous studies showed significant correlations between FGF-23 levels to LV remodeling in patients with LVH [11, 30].

Whereas there are numerous studies examining the association of FGF-23 expression and parameters of LV remodeling, there is a paucity of evidence on the association between FGF-23 and RV remodeling. In a small cohort of PAH patients (*n* = 48) FGF-23 levels were correlated to mPAP, CI, PVR, NT-proBNP und REVEAL Risiko-Score [1]. Data on associations between FGF-23 levels and RV remodeling in PH from bigger cohorts is not available.

In the present study, echocardiographic and RHC parameters were used to detect signs of RV maladaptation and RV-PA uncoupling in PH patients and to analyze their association with FGF-23 levels. Furthermore, associations between FGF-23 levels and parameters of RV afterload were analyzed. Higher FGF-23 concentrations were associated with markedly higher pulmonary pressures and vascular resistance. Notably, tertile analysis showed a consistent relationship between higher FGF-23 levels and worse parameters of RV systolic dysfunction, RV dilation, and RV-PA uncoupling. High FGF-23 levels showed significantly lower TAPSE, TAPSE/PASP, and CI and higher RVEDD. Importantly, high FGF-23 plasma levels in PH were also associated with impaired kidney function. However, patients with normal kidney function showed similar correlations to RV parameters as the entire cohort and regression analysis in the whole PH cohort revealed that FGF-23 is independently associated with parameters of RV function and structure as well as pulmonary pressure and vascular resistance. Previous studies have also shown that high FGF-23 levels in CKD were independently associated with PH, LVH, chronic heart failure, and higher mortality [5, 20, 23].

TAPSE is an established parameter of RV systolic function recommended by the current guidelines, and TAPSE < 17 mm is the cut-off value for systolic dysfunction [7, 29]. The CI was shown to be an independent predictor of cardiac outcome in patients with heart failure [13, 24]. The current guidelines recommend the use of the CI in the prognostic stratification of patients with PH, and CI < 2.5 L/min is associated with increased 1-year mortality [7]. Consequently, TAPSE < 17 mm and CI < 2.5 L/min were used in the present study as cut-offs for systolic dysfunction and low cardiac output to define RV maladaptation with signs of RV-PA uncoupling. In the ROC analysis, FGF-23 was a good predictor of TAPSE < 17 mm and CI < 2.5 L/min. Hence, in our study FGF-23 is an eGFR-independent predictor of maladaptive RV remodeling.

Interestingly, our analysis did not show any significant differences between FGF-23 levels in patients with LVH, DCM, and PH. We assume, that the occurrence of cardiac fibrosis in all three diseases [3, 6, 18] may be the reason for the elevation of FGF-23 levels in all three groups of patients.

Fibrosis appears to be prognostically relevant in RV maladaptation. In PH patients with right heart failure, CMR imaging and histological analyses show a significantly increased amount of RV fibrosis compared with controls [4, 6]. Several studies show that RV fibrosis is associated with RV diastolic dysfunction and adverse outcomes in PH patients [16, 26, 34]. Recently published CMR strain PV loop analyses show that the impaired diastolic function caused by RV fibrosis and intrinsic changes in the cardiomyocytes may be crucial events in the development of RV uncoupling in PH [26, 33]. Therefore, FGF-23 may be a biomarker of RV fibrosis that can detect the transition from adaptive RV hypertrophy to maladaptive RV remodeling and RV-PA uncoupling.

### Limitations

The present clinical data are derived from an observational study without long-term follow-up. Patients included were mainly those with CTEPH and a smaller group with IPAH. Observed associations may therefore not be valid for other PH entities. No core lab analysis of the echocardiographic data was performed. PH group was larger than other groups, which could influence the comparison between the groups. Data on NT-pro-BNP were only available for CTEPH patients; Furthermore, serum levels of calcium, phosphorus, parathormone, and vitamin D are missing, so a possible effect of these parameters on the association between FGF-23 and RV maladaptation could not be detected.

## Conclusion

This study reveals that FGF-23 plasma levels are significantly higher in PH patients than in healthy controls. No differences in FGF-23 concentrations were found between patients with PH, LVH, or DCM. Our analysis also showed that increased FGF-23 plasma levels are associated with systolic RV dysfunction, RV dilation, and higher pulmonary pressures in PH patients. Furthermore, FGF-23 was a good predictor of RV maladaptation and may thus serve as a biomarker for maladaptive RV remodeling in patients with PH.

### Supplementary Information

Below is the link to the electronic supplementary material.

Supplementary file1 (DOCX 13 kb)

Supplementary file2 (DOCX 14 kb)

## Data Availability

The data that support the findings of this study are available from the corresponding author, SK, upon reasonable request.

## Abbreviations

FGF-23: Fibroblast growth factor 23
RV-PA coupling: Right ventricular to pulmonary artery coupling
NT-pro-BNP: N-terminal pro-brain natriuretic peptide
CAD: Coronary artery disease
DCM: Dilated cardiac myopathy
ECM: Extracellular matrix
LV: Left ventricular
RV: Right ventricular
RVEDD: Right ventricular end diastolic diameter
LVH: Left ventricular hypertrophy
LV-EF: Left ventricular ejection fraction
mPAP: Mean pulmonary artery pressure
PASP: Systolic pulmonary artery pressure
TAPSE: Tricuspid annular plane systolic excursion
PH, PAH: Pulmonary hypertension, pulmonary arterial hypertension
IPAH: Idiopathic pulmonary arterial hypertension
PVR: Pulmonary vascular resistance
CI: Cardiac index
RAP: Right atrial pressure
CTEPH: Chronic thromboembolic pulmonary hypertension

## References

[1] Bouzina H, Hesselstrand R, Radegran G (2019). Higher plasma fibroblast growth factor 23 levels are associated with a higher risk profile in pulmonary arterial hypertension. Pulm Circ.

[2] Campo A, Mathai SC, Le Pavec J, Zaiman AL, Hummers LK, Boyce D, Housten T, Lechtzin N, Chami H, Girgis RE, Hassoun PM (2011). Outcomes of hospitalisation for right heart failure in pulmonary arterial hypertension. Eur Respir J.

[3] Cojan-Minzat BO, Zlibut A, Agoston-Coldea L (2021). Non-ischemic dilated cardiomyopathy and cardiac fibrosis. Heart Fail Rev.

[4] Drake JI, Bogaard HJ, Mizuno S, Clifton B, Xie B, Gao Y, Dumur CI, Fawcett P, Voelkel NF, Natarajan R (2011). Molecular signature of a right heart failure program in chronic severe pulmonary hypertension. Am J Respir Cell Mol Biol.

[5] Faul C, Amaral AP, Oskouei B, Hu M-C, Sloan A, Isakova T, Gutiérrez OM, Aguillon-Prada R, Lincoln J, Hare JM (2011). FGF23 induces left ventricular hypertrophy. J Clin Investig.

[6] Freed BH, Gomberg-Maitland M, Chandra S, Mor-Avi V, Rich S, Archer SL, Jamison EB, Lang RM, Patel AR (2012). Late gadolinium enhancement cardiovascular magnetic resonance predicts clinical worsening in patients with pulmonary hypertension. J Cardiovasc Magn Reson Off J Soc Cardiovasc Magn Reson.

[7] Galie N, Humbert M, Vachiery JL, Gibbs S, Lang I, Torbicki A, Simonneau G, Peacock A, Vonk Noordegraaf A, Beghetti M, Ghofrani A, Gomez Sanchez MA, Hansmann G, Klepetko W, Lancellotti P, Matucci M, McDonagh T, Pierard LA, Trindade PT, Zompatori M, Hoeper M (2016). 2015 ESC/ERS guidelines for the diagnosis and treatment of pulmonary hypertension. Rev Esp Cardiol.

[8] Grabner A, Amaral AP, Schramm K, Singh S, Sloan A, Yanucil C, Li J, Shehadeh LA, Hare JM, David V, Martin A, Fornoni A, Di Marco GS, Kentrup D, Reuter S, Mayer AB, Pavenstadt H, Stypmann J, Kuhn C, Hille S, Frey N, Leifheit-Nestler M, Richter B, Haffner D, Abraham R, Bange J, Sperl B, Ullrich A, Brand M, Wolf M, Faul C (2015). Activation of cardiac fibroblast growth factor receptor 4 causes left ventricular hypertrophy. Cell Metab.

[9] Gruson D, Lepoutre T, Ketelslegers JM, Cumps J, Ahn SA, Rousseau MF (2012). C-terminal FGF23 is a strong predictor of survival in systolic heart failure. Peptides.

[10] Guazzi M, Bandera F, Pelissero G, Castelvecchio S, Menicanti L, Ghio S, Temporelli PL, Arena R (2013). Tricuspid annular plane systolic excursion and pulmonary arterial systolic pressure relationship in heart failure: an index of right ventricular contractile function and prognosis. Am J Physiol Heart Circ Physiol.

[11] Han J, Yuan X, Song W, Cheng Y, Lu Y, Zhang Y, Liu Y, Jiang Y (2022). The correlation of fibroblast growth factor 23 with cardiac remodeling in essential hypertension with normal renal function. Cardiology.

[12] Hao H, Li X, Li Q, Lin H, Chen Z, Xie J, Xuan W, Liao W, Bin J, Huang X, Kitakaze M, Liao Y (2016). FGF23 promotes myocardial fibrosis in mice through activation of β-catenin. Oncotarget.

[13] Ibe T, Wada H, Sakakura K, Ugata Y, Maki H, Yamamoto K, Seguchi M, Taniguchi Y, Jinnouchi H, Fujita H (2021). Cardiac index predicts long-term outcomes in patients with heart failure. PLoS One.

[14] Isakova T, Gutierrez O, Shah A, Castaldo L, Holmes J, Lee H, Wolf M (2008). Postprandial mineral metabolism and secondary hyperparathyroidism in Early CKD. J Am Soc Nephrol.

[15] Isakova T, Houston J, Santacruz L, Schiavenato E, Somarriba G, Harmon WG, Lipshultz SE, Miller TL, Rusconi PG (2013). Associations between fibroblast growth factor 23 and cardiac characteristics in pediatric heart failure. Pediatr Nephrol.

[16] Keranov S, Dorr O, Jafari L, Troidl C, Liebetrau C, Kriechbaum S, Keller T, Voss S, Bauer T, Lorenz J, Richter MJ, Tello K, Gall H, Ghofrani HA, Mayer E, Wiedenroth CB, Guth S, Lorchner H, Poling J, Chelladurai P, Pullamsetti SS, Braun T, Seeger W, Hamm CW, Nef H (2021). CILP1 as a biomarker for right ventricular maladaptation in pulmonary hypertension. Eur Respir J.

[17] Lang RM, Badano LP, Mor-Avi V, Afilalo J, Armstrong A, Ernande L, Flachskampf FA, Foster E, Goldstein SA, Kuznetsova T, Lancellotti P, Muraru D, Picard MH, Rietzschel ER, Rudski L, Spencer KT, Tsang W, Voigt JU (2015). Recommendations for cardiac chamber quantification by echocardiography in adults: an update from the American Society of Echocardiography and the European Association of Cardiovascular Imaging. Eur Heart J Cardiovasc Imaging.

[18] Lazzeroni D, Rimoldi O, Camici PG (2016). From left ventricular hypertrophy to dysfunction and failure. Circ J.

[19] Leifheit-Nestler M, Haffner D (2018). Paracrine effects of FGF23 on the heart. Front Endocrinol.

[20] Miri M, Ahmadi M, Hatami M (2021). Correlation Between fibroblast growth factor-23 and pulmonary arterial hypertension in hemodialysis patients. Iran J Kidney Dis.

[21] Mirza MA, Larsson A, Melhus H, Lind L, Larsson TE (2009). Serum intact FGF23 associate with left ventricular mass, hypertrophy and geometry in an elderly population. Atherosclerosis.

[22] Naeije R, Manes A (2014). The right ventricle in pulmonary arterial hypertension. Eur Respir Rev Off J Eur Respir Soc.

[23] Niizuma S, Iwanaga Y, Yahata T, Miyazaki S (2017). Renocardiovascular biomarkers: from the perspective of managing chronic kidney disease and cardiovascular disease. Front Cardiovasc Med.

[24] Patel CB, DeVore AD, Felker GM, Wojdyla DM, Hernandez AF, Milano CA, O'Connor CM, Rogers JG (2014). Characteristics and outcomes of patients with heart failure and discordant findings by right-sided heart catheterization and cardiopulmonary exercise testing. Am J Cardiol.

[25] Plischke M, Neuhold S, Adlbrecht C, Bielesz B, Shayganfar S, Bieglmayer C, Szekeres T, Horl WH, Strunk G, Vavken P, Pacher R, Hulsmann M (2012). Inorganic phosphate and FGF-23 predict outcome in stable systolic heart failure. Eur J Clin Investig.

[26] Rain S, Handoko ML, Trip P, Gan CT, Westerhof N, Stienen GJ, Paulus WJ, Ottenheijm CA, Marcus JT, Dorfmuller P, Guignabert C, Humbert M, Macdonald P, Dos Remedios C, Postmus PE, Saripalli C, Hidalgo CG, Granzier HL, Vonk-Noordegraaf A, van der Velden J, de Man FS (2013). Right ventricular diastolic impairment in patients with pulmonary arterial hypertension. Circulation.

[27] Rodelo-Haad C, Santamaria R, Muñoz-Castañeda JR, Pendón-Ruiz de Mier MV, Martin-Malo A, Rodriguez M (2019). FGF23, biomarker or target?. Toxins.

[28] Roy C, Lejeune S, Slimani A, de Meester C, Ahn As SA, Rousseau MF, Mihaela A, Ginion A, Ferracin B, Pasquet A, Vancraeynest D, Beauloye C, Vanoverschelde J-L, Horman S, Gruson D, Gerber BL, Pouleur A-C (2020). Fibroblast growth factor 23: a biomarker of fibrosis and prognosis in heart failure with preserved ejection fraction. ESC Heart Fail.

[29] Rudski LG, Lai WW, Afilalo J, Hua L, Handschumacher MD, Chandrasekaran K, Solomon SD, Louie EK, Schiller NB (2010). Guidelines for the echocardiographic assessment of the right heart in adults: a report from the American Society of Echocardiography endorsed by the European Association of Echocardiography, a registered branch of the European Society of Cardiology, and the Canadian Society of Echocardiography. J Am Soc Echocardiogr Off Publ Am Soc Echocardiogr.

[30] Shibata K, Fujita S, Morita H, Okamoto Y, Sohmiya K, Hoshiga M, Ishizaka N (2013). Association between circulating fibroblast growth factor 23, alpha-Klotho, and the left ventricular ejection fraction and left ventricular mass in cardiology inpatients. PLoS One.

[31] Shimada T, Hasegawa H, Yamazaki Y, Muto T, Hino R, Takeuchi Y, Fujita T, Nakahara K, Fukumoto S, Yamashita T (2004). FGF-23 is a potent regulator of vitamin D metabolism and phosphate homeostasis. J Bone Miner Res Off J Am Soc Bone Miner Res.

[32] Tello K, Axmann J, Ghofrani HA, Naeije R, Narcin N, Rieth A, Seeger W, Gall H, Richter MJ (2018). Relevance of the TAPSE/PASP ratio in pulmonary arterial hypertension. Int J Cardiol.

[33] Tello K, Dalmer A, Vanderpool R, Ghofrani HA, Naeije R, Roller F, Seeger W, Wilhelm J, Gall H, Richter MJ (2019). Cardiac magnetic resonance imaging-based right ventricular strain analysis for assessment of coupling and diastolic function in pulmonary hypertension. JACC Cardiovasc Imaging.

[34] Trip P, Rain S, Handoko ML, van der Bruggen C, Bogaard HJ, Marcus JT, Boonstra A, Westerhof N, Vonk-Noordegraaf A, de Man FS (2015). Clinical relevance of right ventricular diastolic stiffness in pulmonary hypertension. Eur Respir J.

[35] van der Bruggen CEE, Tedford RJ, Handoko ML, van der Velden J, de Man FS (2017). RV pressure overload: from hypertrophy to failure. Cardiovasc Res.

[36] Vonk-Noordegraaf A, Haddad F, Chin KM, Forfia PR, Kawut SM, Lumens J, Naeije R, Newman J, Oudiz RJ, Provencher S, Torbicki A, Voelkel NF, Hassoun PM (2013). Right heart adaptation to pulmonary arterial hypertension: physiology and pathobiology. J Am Coll Cardiol.

[37] Vonk Noordegraaf A, Westerhof BE, Westerhof N (2017). The relationship between the right ventricle and its load in pulmonary hypertension. J Am Coll Cardiol.

