# Supplementary material for: Fibroblast growth factor 23 as a biomarker of right ventricular dysfunction in pulmonary hypertension
Source: Clin Res Cardiol. 2023 Feb 15;112(10):1382–93. doi: 10.1007/s00392-023-02162-y (PMC10562503; doi:10.1007/s00392-023-02162-y)
Supplement: Supplementary file 1 — Supplementary file1 (DOCX 13 kb) [file 392_2023_2162_MOESM1_ESM.docx]

| **Target variable** | **predictor** | **p-value** | **OR** | **95% Confidence Interval** |
| --- | --- | --- | --- | --- |
| TAPSE < 17 mm | FGF-23 | <0.001 | 1.21 | 1.09-1.34 |
|  | GFR | 0.21 | 1.00 | 0.99-1.00 |
| CI < 2.0 L/min/m^2^ | FGF-23 | 0.01 | 1.13 | 1.04-1.24 |
|  | GFR | 0.98 | 1.00 | 0.99-1.01 |
| RVEDd > 43mm | FGF-23 | 0.05 | 1.44 | 1.00-2.08 |
|  | GFR | 0.82 | 1.00 | 0.99-1.01 |
| RAP > 7mmHg | FGF-23 | <0.001 | 1.51 | 1.18-1.92 |
|  | GFR | 0.77 | 1.00 | 0.99-1.01 |

Suppl. Table 1: Binary logistic regression

OR, Odds Ratio; TAPSE, tricuspidal annular plane systolic excursion; CI, cardiac index; RVEDd, right ventricular enddiastolic diameter; RAP, right atrial pressure
