# Supplementary material for: Fibroblast growth factor 23 as a biomarker of right ventricular dysfunction in pulmonary hypertension
Source: Clin Res Cardiol. 2023 Feb 15;112(10):1382–93. doi: 10.1007/s00392-023-02162-y (PMC10562503; doi:10.1007/s00392-023-02162-y)
Supplement: Supplementary file 2 — Supplementary file2 (DOCX 14 kb) [file 392_2023_2162_MOESM2_ESM.docx]

**A**

|  | **Pearson correlation** | **p-value** |
| --- | --- | --- |
| **age** | 0.03 | ns |
| **GFR** | -0.33 | <0.001 |
| **RVEDD** | 0.32 | <0.001 |
| **TAPSE** | -0.30 | <0.01 |
| **TAPSE/PASP** | -0.37 | <0.01 |
| **LVEF** | 0.05 | ns |
| **LVEDD** | -0,28 | 0,001 |
| **LVPWd** | 0.02 | ns |
| **IVSd** | -0.08 | ns |
| **mPAP** | 0.28 | <0.001 |
| **PAWPmean** | 0.05 | ns |
| **RAP** | 0.32 | 0.005 |
| **CI** | -0.20 | <0.001 |
|  |  |  |

**B**

|  | **Pearson correlation** | **p-value** |
| --- | --- | --- |
| **age** | -0.15 | ns |
| **GFR** | -0.11 | ns |
| **RVEDD** | 0.59 | <0.001 |
| **TAPSE** | -0.31 | <0.01 |
| **TAPSE/PASP** | -0.39 | <0.01 |
| **LVEF** | -0.94 | ns |
| **LVEDD** | -0.42 | <0.001 |
| **LVPWd** | 0.02 | ns |
| **IVSd** | -0.10 | ns |
| **mPAP** | 0.17 | ns |
| **PAWPmean** | 0.05 | ns |
| **RAP** | 0.37 | 0.005 |
| **CI** | -0.28 | 0.01 |
|  |  |  |

Suppl. Table 2: Correlation of FGF-23 with clinical, echocardiographic and invasively measured characteristics in A) the entire PH cohort and in B) PH patients with CKD (GFR<60 mL/min/1.73m2 )

FGF-23, fibroblast growth factor 23; CKD, chronic kidney disease; GFR, glomerular filtration rate; RVEDD, right ventricular enddiastolic diameter; TAPSE, tricuspid annular plane systolic excursion; PASP, pulmonary artery systolic pressure; LVEF, left-ventricular ejection fraction; LVEDD, left ventricular enddiastolic diameter; LVPWd, diastolic left ventricular posterior wall thickness; IVSd, diastolic interventricular septum thickness; mPAP, mean pulmonary artery pressure, PAWPmean, mean pulmonary artery wedge pressure; RAP, right atrial pressure; CI, cardiac index
